# Perception of animal welfare issues during Chinese transport and slaughter of livestock by a sample of stakeholders in the industry

**DOI:** 10.1371/journal.pone.0197028

**Published:** 2018-06-22

**Authors:** Xiaofei Li, Sarah Zito, Michelle Sinclair, Clive J. C. Phillips

**Affiliations:** Centre for Animal Welfare and Ethics, School of Veterinary Science, University of Queensland, Gatton, Queensland, Australia; Gaziosmanpasa University, TURKEY

## Abstract

China is the world’s biggest livestock producer, and has a rapidly expanding intensive livestock production in response to growing demand. The large size of the country and geographical dispersion of the livestock production systems means that animals are often transported long distances to slaughter. This study investigated perceptions of animal welfare issues by stakeholders in the Chinese transport and slaughter industry using utility scores and adaptive conjoint analysis. An initial workshop for experts in this field identified key concerns; these were then included in a questionnaire, which was distributed electronically to stakeholders. Stakeholders, particularly those with higher levels of education, were most concerned about the absence of pre-slaughter stunning and failure to maintain unconsciousness throughout the slaughter process. For all livestock species electrical stunning was considered the best method of stunning and blunt trauma the worst; for cattle and sheep stunning using a penetrating captive bolt was considered preferable to the use a percussive captive bolt. Other concerns considered very important were journey quality and livestock workers’ experience and attitudes. Heat stress and closed-sided vehicles were of greater concern than cold stress. Loading facilities and journey length were considered of intermediate importance, while lairage and methods for catching chickens were of least concern. The importance of some welfare concerns, e.g. livestock having to remain standing during a journey, was more commonly recognised by stakeholders who reported a high level of knowledge and experience. Therefore, these welfare issues could be a focus for future training activities. Compared to respondents directly involved in livestock transport, respondents involved in teaching and researching within livestock production rated the presented animal welfare issues as more important. These results can be used to guide development of training programmes, animal welfare research, and certification and regulatory control to target challenges to animal welfare in livestock transport and slaughter in China.

## Introduction

The livestock industry in the People’s Republic of China (hereafter referred to as China) has rapidly developed over the past two decades [1]. In 2014, China produced approximately five billion chickens, 480 million pigs, 202 million sheep and 117 million cattle, making it the world’s largest livestock producer [1]. It was the world’s biggest meat producer (approximately 83 billion tonnes in 2013) [2], with major exports of beef [3], pork [4], chicken and other meats [4]. In addition, its population is one of the world’s largest meat consumers [5]. Therefore, the livestock industry is of considerable economic importance in China, with an estimated output value of RMB 25.8 trillion (approximately 4 trillion USD) in 2011, which was 31.7% of the total value of Chinese agriculture (including crops, forestry, livestock, and fishery) [6].

The scale of livestock production and its associated intensification has resulted in growing animal welfare concerns, both in terms of numbers of animals and the welfare conditions [7, 8]. Chinese traditional ethics from Confucianism, Taoism, and Buddhism support compassion for non-human animals [8]. However, with the current industrialisation of China’s livestock production, many Western farming practices (such as gestation crates, castration, tail docking and ear-clipping of pigs, beak-trimming and battery cages for hens, castration and early weaning of calves, and forced feeding of ducks and geese for foie-gras production) are being adopted and utilised with increasing frequency. At the same time, some Western countries (particularly in the European Union) are phasing out some of these practices, such as battery cages and stalls for sows during gestation [9]. Due to the large numbers of livestock produced in China, this is probably one of the major animal welfare concerns in the world today [8]. Within China the concept of animal welfare is little understood, and in a recent survey of public opinions toward farm animal welfare in China, approximately two thirds of respondents did not know what the term meant [10]. In Chinese, the usual translation is ‘dongwu fuli’. Fuli literally means interests in life, especially care taken for employees by providing food, shelter and medical care, thus creating a concept of a high level of provision for needs which has then been applied to animals (dongwu)[11]. The high level of provision described in this concept may lead some to question the validity of concern for animal welfare, when humans are not afforded all of these provisions. Confusion also exists between animal welfare and animal rights causes [12]. Motivation in China to improve animal welfare has been lacking due to concern that farm profitability would be adversely affected as a consequence [8, 11]. Nevertheless, there is a growing recognition that trade with overseas partners will require international animal welfare standards to be met [8, 11, 12, 13, 14]. China’s trade partners in both the import and export markets have expressed concern regarding Chinese animal welfare standards [15, 16, 17]. However, there are an increasing number of Chinese animal protectionists, and they are one of the most active interest groups in China [8]. The proposal, in 2009, to introduce a Prevention of Cruelty to Animals Law in China was an important development in animal welfare in the country, but the progress on advancing animal welfare has been slow and there is still no national farm animal protection legislation in effect [11, 12, 13, 14].

In terms of the regulations or legislations related to animal welfare in China, a pig slaughtering management regulation was first introduced in 1998 and in December 2007 it was revised [18], but neither of these required stunning before slaughter. In 2008, an act outlining requirements for humane slaughter was drafted [12]. Since 2014, there have been several proposals to develop more comprehensive livestock regulations for animal welfare, posed by for example the China Association for Standardization [19], the Chinese Veterinary Medical Association, and in 2017 the Chinese government [12].

The welfare of animals in livestock industries is a responsibility shared between farmers, the livestock companies and industry service providers, such as transporters, slaughter facilities, and veterinarians [20]. Livestock industries must comply with animal welfare legislation but are also indirectly responsible to the public and consumers for the welfare of the farm animals [20]. Consumers in many countries have shown increasing interest in animal welfare issues in recent years, and improvements in animal welfare standards have often been driven by concern from consumers and the public [21]. As well as putting pressure on industry to improve farm animal welfare, consumers are increasingly willing to choose products based at least partly on the perceived welfare of the animals [10, 20, 21]. For example, consumers in Shanghai and Beijing are now showing a preference for pork with better animal welfare claimed in the labelling, not just because they thought it was tastier and safer but for the benefit of the animals [22]. Successful management of issues with a human dimension requires involvement of stakeholders in the definition of the problem to be addressed, decision-making, and development of relevant and targeted strategies informed by research [23, 24, 25]. The importance of understanding and engaging local stakeholders in strategies to address issues has been demonstrated in other comparable areas of human-animal interaction, such as human-wildlife conflict, wildlife and environmental conservation, and management of introduced animals [23, 26]. Marketing theory stresses the importance of understanding the target audience in order to make marketing campaigns relevant and engaging [27]. Engaging stakeholders in the Chinese livestock system to improve animal welfare first requires an understanding of what the perceived welfare issues are, but they are little understood for Chinese livestock transport and slaughter.

Livestock transport and slaughter are two areas in which animal welfare may be of considerable concern. Animals in China are transported over long distances (for example, chickens travelling 2500 km from Jilin, near North Korea, to Shenzhen, just north of Hong Kong [28]), which could potentially have serious adverse effects on their welfare [28]. In addition, humane slaughter is a relatively new concept in China, with currently no requirements for this in many slaughter facilities [8, 10].

Choice tests can be used to gauge stakeholders’ attitudes to animal welfare, in which they select which of two options provides the better welfare for animals [20, 29, 30]. Such conjoint analysis can be adaptive (Adaptive Conjoint Analysis, ACA), in which case a computer customizes the questions according to the respondents’ answers, giving advantages over traditional survey techniques [20]. This technique was designed for market research to determine consumers’ product preferences [31, 32] but has also been adapted to determine opinions related to the importance of different welfare issues in Australian livestock production [20,33]. The strength of this technique is its ability to ask respondents realistic questions which mimic the trade-offs that people need to make in the real world. For example, it is not always possible to ensure that every aspect of animal welfare is ideal in a given scenario. Therefore, it is necessary to decide which aspect of an animal’s welfare is most important so that it can be prioritized. Web-based interactive ACA questionnaires can be constructed using intuitive software (e.g. Sawtooth Software Inc., Sequim, WA, USA), which allows emphasis on issues that require additional questions because previous responses are too similar to indicate the respondent’s preferences. This adaptive capacity facilitates a rapid and accurate definition of the preferences or opinions of the questionnaire respondent [33]. The computer generates utility values, which are measures of the respondent’s relative strength of preference for each level of welfare provision on each issue. For example, for the welfare issue of stunning before slaughter, the levels might be electrical stunning, penetrating captive bolt, percussive captive bolt, blunt trauma and no stunning. In our opinion, a web-based interactive ACA questionnaire was a better technique for the purposes of this study than a conventional questionnaire.

The objectives of this study were to establish what the perceived animal welfare issues in livestock slaughter and transport are in China, using ACA and utility scores, analysed through multivariable analyses. When coupled with *in situ* measurements of welfare, this information is anticipated to improve understanding of animal welfare issues in transport and slaughter in the Chinese livestock industry and inform the direction of future animal welfare improvement.

## Material and methods

### Ethics statement

The study was approved by the University of Queensland Ethics Committee (Project No.2015001840).

### Establishing the welfare issues

An initial one-day focus group meeting in December 2015 addressed the question ‘what are the most readily identified farm animal welfare concerns for commercial animal welfare professionals in China?’ Participants excluded fish as a farm animal of interest. The participants consisted of eight senior livestock transport and slaughter company representatives and livestock production academics operating in and around Guangzhou, China. They were invited by a senior academic representative of South China Agricultural University based on their pre-eminence in the livestock sector. The participants also had self-identified interests in improving animal welfare for commercial reasons, including gaining access to export markets. The area of Guangzhou was chosen because it is one of the most important regions in China in terms of livestock production, including that supported by foreign investment [34]. Attendance was voluntary and unrewarded.

In the focus group discussion, which took the form of a wide-net participant-directed brainstorm, respondents systematically commented on potential welfare challenges within their industries. After discussing the issues generally in the first half of the day, the group was asked to collectively decide on the 15 most pressing animal welfare concerns during livestock transport and slaughter in China. The major issues were listed, and discussed again to ensure that the list was an accurate representation of their collective view.

The issues identified for livestock during transport were:

1) Experience of livestock transport workers
2) Attitude of livestock transport workers
3) Method of catching chickens
4) Part of body used to catch fully developed chickens
5) Livestock loading facilities
6) Type of road vehicle used to transport livestock
7) Overcrowding of livestock in transport vehicles
8) Length of journey to slaughterhouse
9) Maintenance of animal comfort during transportation (temperature)
10) Maintenance of animal comfort during transportation (provision of rest and water)
11) Stress to livestock during journey

Issues identified for livestock during slaughter were:

1) Pre-slaughter accommodation
2) Stunning procedures for poultry and pigs
3) Stunning procedures for cattle and sheep
4) Achievement and maintenance of unconsciousness during the slaughter process

Water injecting of pigs was raised as a potential issue although it was not included in the distributed list as it is currently illegal in China. The practice involves forcing water into the stomach of pigs shortly before slaughter, or injecting it into their hearts, to increase their weight. Each issue was further described by two to five levels (or different livestock management scenarios) with different welfare implications (Table 1). These were developed and refined during the meeting.

**Table 1:** Welfare issues and their utility components, indicating the relative strength of preference for each level of welfare provision on each issue. Each issue had 2–5 utility levels demonstrating the perceived importance for animal welfare. The issues are listed in declining order of importance value (the difference that each issue made to the total of all the utility values for each respondent). The average importance and zero-centred utility values are listed for each issue, as rated by respondents (n = 267), who were stakeholders in the animal transportation and slaughter sectors, to a questionnaire on the major welfare issues in the Chinese livestock transport and slaughter industry.

Issue and its importance value	Levels	Utility value
Stunning procedures for cattle and sheep (8.64)	Electrically stunned before they are slaughtered	36.3
	Stunned with a penetrating captive bolt	9.9
	Stunned with a percussive captive bolt	-4.5
	Not stunned before they are slaughtered	-13.3
	Stunned by hitting them on the head with a hard object	-28.4
Stunning procedures for pigs and poultry (8.52)	Electrically stunned before they are slaughtered	27.1
	Stunned with carbon dioxide before they are slaughtered	24.8
	Not stunned before they are slaughtered	-13.2
	Stunned by hitting them on the head with a hard object	-38.7
Stress to livestock during journey (7.78)	Comfortable journey with no evidence of bruising or vomiting	53.1
	Uncomfortable and stressful journey and there is some evidence of bruising or vomiting	-12.8
	Stressful journey and there is significant bruising and mortality of livestock	-40.3
Maintenance of animal comfort during transportation (temperature) (7.72)	Comfortable for the livestock	55.3
	Causes cold stress to the livestock	-20.5
	Causes heat stress to the livestock	-34.8
Achievement and maintenance of unconsciousness during the slaughter process (7.15)	Remain unconscious throughout the slaughter process	28.9
	Regain consciousness after stunning during the slaughter process	-28.9
Experience of livestock transport workers (7.06)	High	39.4
	Moderate	5.6
	Little	-45.0
Attitude of livestock transport workers (6.47)	Good	30.0
	Reasonable	12.9
	Poor	-42.8
Overcrowding of livestock in transport vehicles (6.26)	Not able to stand up due to overcrowding	34.3
	Able to stand up	-34.3
Type of road vehicle (6.19)	Open-sided vehicle	16.5
	Semi-closed vehicle	10.5
	Closed-sided vehicle	-26.9
Maintenance of animal comfort during transportation (provision of rest and water) (6.19)	Provided with a stop for water	26.7
	Not provided with a stop for water	-26.7
Loading facilities (5.85)	With a gently sloping loading ramp, a non-slip floor and no sharp turns	17.2
	With a steep loading ramp slope, a slippery floor or sharp turns	-17.2
Duration of journey to slaughterhouse (5.70)	Less than 3 hours	27.7
	3–6 hours	0.6
	More than 6 hours	-28.3
Pre-slaughter accommodation for cattle, pigs and sheep (5.59)	Provided with 6 hours or more rest and water before slaughter	28.3
	Provided with no rest or water before slaughter	-28.3
Method of catching chickens (5.51)	Manually in the light	18.2
	Manually in the dark	-18.2
Part of chicken’s body used for catching (5.38)	Manually caught by the legs and feet	30.0
	Manually caught by the head or wings or tail	-30.0

After the focus group meeting, two new issues were added by the authors of this paper

to ensure that the issues were balanced and consistent: ‘attitude of livestock transport workers’ and ‘level of experience of livestock transport workers’. In addition, the ‘maintenance of animal comfort during transportation through provision of rest and water’ was added to ensure clarity and consistency with a question about stress, and ‘stunning procedure for cattle and sheep’ was added to match the issue that was raised for pigs in the focus group meeting.

### The questionnaire (S1 and S2 Tables)

The outcome of this focus group was used as the basis for a survey using intuitive software, which was distributed electronically to a wider group of stakeholders in China, to include a more diverse work force within the animal transportation and slaughter sectors and increase the number of respondents. The questionnaire was developed using the list of issues, relevant literature, and consultation with industry and academic stakeholders. Using a web-based interactive ACA questionnaire format, it was piloted with volunteers and changes were made based on their feedback. After finalising the questionnaire in English, it was translated into Mandarin by a professional translator. The meaning and translation were checked for accuracy and consistency of meaning by a third party fluent in both Mandarin and English. In addition, our academic partners in China were sent both the English and Mandarin versions of the questionnaire to compare in order to double check the accuracy of the questionnaire translation.

The finalised questionnaire was then loaded into the online software (Sawtooth Software version 8.4.8., Sawtooth Software Inc., Sequim, WA, USA). This has the advantages of being able to intelligently adapt the questionnaire in real-time to each respondent based on their answers and engaging the respondent by using a computer-interactive method [33].

#### Distribution of the questionnaire to potential respondents

The questionnaire was distributed to people involved in livestock transport and slaughter in the China during February and March 2016. We used a virtual snowballing technique, starting with our academic and industry partners and stakeholders in the Chinese livestock industry. A recruitment e-mail with information about the study was sent to the focus group attendees; they were then asked to forward the recruitment email to all Chinese livestock industry stakeholders in their social and professional networks with request for them in turn to forward the recruitment e-mail to other relevant people. A link in the e-mail connected them directly to the survey site. Those who volunteered to participate clicked on the link and were directed to the first page of the survey, where participants were shown information about the questionnaire. This stated that: participation was voluntary, participants needed to be 18 years of age or older, the study had been cleared in accordance with the ethical review guidelines and processes of The University of Queensland, and that participants could discuss their participation with project staff or the School Ethics officer (contact details were given). Respondents confirmed on-line that they were over 18 years of age, that they understood that their participation in the study was voluntary, consented to participate, and to their responses being used as outlined. Only participants who gave consent answered questions from the survey. Demographic questions were used to gain information about type and duration of the Chinese livestock industry, gender, age, education level, geographical location of work relating to the Chinese livestock industry, and knowledge of livestock transport and slaughter in China. Finally, respondents were provided with a definition of animal welfare (based on the World Organisation for Animal Health definition in the Terrestrial Code [35]): “Animal welfare means how an animal is coping with the conditions in which it lives. An animal is in a good state of welfare if it is healthy, comfortable, well-nourished, safe, able to express innate behaviour, and if it is not suffering from unpleasant states such as pain, fear, and distress.”

#### The sections of the questionnaire, including adaptive conjoint analysis

**Part 1: Rating of levels.** First, issue levels were screened by asking the respondent to rate the importance of each, which allowed the software to learn enough about the respondent’s preferences to construct initial part-worth estimates. Respondents were given an introduction before they were presented with the issues: “In this section you will be asked about different livestock management scenarios and, in your opinion, how acceptable they are in relation to animal welfare. Please consider only the immediate impact of each scenario on the animals’ welfare, regardless of the associated practical and economic factors.” The responses were recorded on a 5-point Likert scale from “not acceptable” to “very acceptable”.

**Part 2: Importance questions.** Respondents were asked to rate how important they thought the difference was between their perceived worst and best levels of animal welfare for each of 15 issues (Table 1). They were given an introduction before they were presented with the perceived best and worst levels for each issue: “In this next section, you will be presented with two livestock management scenarios. Please consider whether the scenarios are different in terms of their immediate impact on animal welfare. If you think that animal welfare does not differ between the two scenarios, please choose ‘not important’. If you think there is a difference between the two scenarios, then use the scale to show how important you think the difference is (please assume that all other aspects of the livestock management are acceptable)”. The 5-point Likert scale was from “not important” to “extremely important”.

For example:

Respondents were asked to rate the importance, in terms of animal welfare, of the difference between an animal remaining unconscious throughout the slaughter process and an animal regaining consciousness during the slaughter process after stunning.

**Part 3: Customized paired comparison trade-off questions.** Respondents were presented with ten sets of two combinations of livestock management scenarios, based on their previous answers. Since it was not practical to have respondents evaluate all possible animal welfare combinations, the ACA approach allowed the efficient collection of detailed information on animal welfare issue importance by presenting respondents with only a carefully chosen set of hypothetical welfare scenarios for evaluation. Questions provided the most additional information by selecting concepts that were known to be nearly equal in importance to the respondent, given what was already known about the respondent's values from their previous responses. The respondent was then asked which of the pairs of hypothetical welfare scenarios was preferable to them assuming that all other aspects of the livestock management were acceptable and considering just the immediate impact of each procedure on the animal welfare. Following each response, the estimates of part-worthy were updated to allow the software to determine what pair of concepts to present to the respondent next. Certain pairs of issue levels that rationally could not appear together in the same concept were specified as part of the questionnaire logic (for example, welfare scenarios that are specific to poultry with a welfare scenario that is specific to cattle). In contrast to direct questioning methods simply asking how important each issue is, conjoint analysis forces respondents to make difficult trade-offs, as in a real world decision-making process [36].

**Fig 1:**
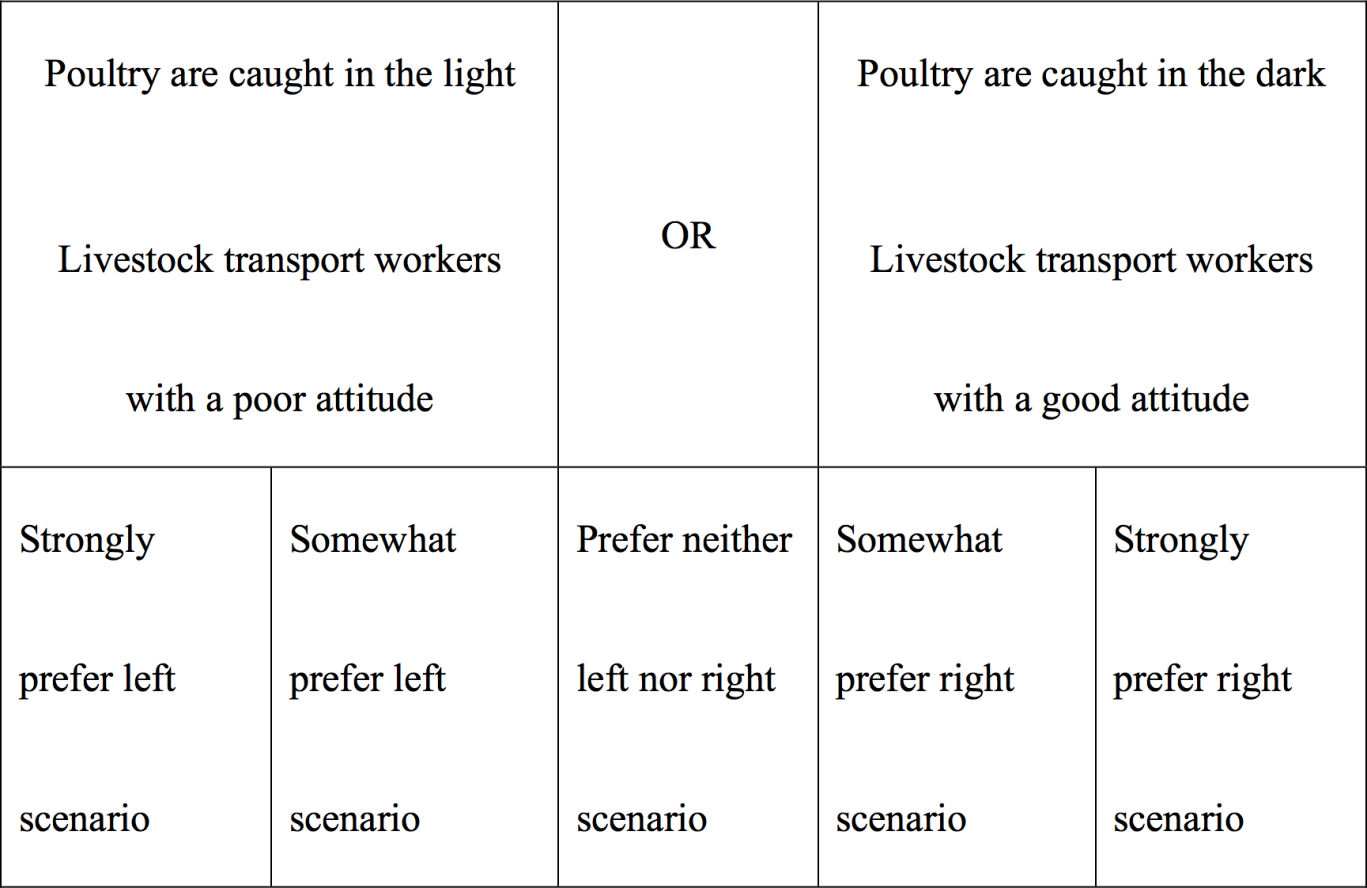
Example of combination scenario.

One combination was presented on the LEFT side of the screen and the other on the RIGHT side (Fig 1).

**Part 4: ACA calibration questions.** Six calibrating scenarios were composed by the software, using those issues determined to be most important to the respondents, who were asked to rate how acceptable the scenario was to them from an animal welfare point of view. They selected a number between 0 (definitely unacceptable from an animal welfare point of view) and 100 (very acceptable from an animal welfare point of view). Scenarios were chosen to occupy the entire range from very unacceptable to very acceptable for the respondent. The concept the respondent was expected to find least acceptable among all possible concepts was presented first, and the one expected to be most acceptable was presented second. Those two concepts established a frame of reference and the remaining concepts were selected to have intermediate levels of acceptability. Each animal welfare scenario had two to five components, for example:

Livestock transport workers with a poor attitude.

Fully developed chickens are manually caught by the legs and feet.

Poultry are caught manually in the light.

Finally, respondents were given the opportunity to comment in a free text field whether there were any other welfare issues associated with livestock transport and slaughter in China that they believed were important but were not covered in the questionnaire.

### Data analysis

#### Utility values

At the end of the ACA questionnaire respondent utility estimates, indicating mean acceptability of each level, were calculated using a hierarchical Bayes utility estimation that estimated strengths of preference for different levels of the welfare issues. The Sawtooth software employed an algorithm to calculate the average utilities for the entire sample [37]. Utility estimates were determined for each Level of each Issue using a simulator in the software, which imported respondents’ Level preferences into a hierarchical Bayes model using a Monte Carlo Markov chain algorithm. This uses data from the population (means and covariances) to estimate the probability distribution of the parameters using conditional probability techniques at different levels [38]. The data were normalised across respondents by zero-centring the Utility values within each Issue so that their sum was equal to zero. Levels of an Issue rated highly by respondents were indicated by a positive Utility value, and those given a low rating, a negative value. The utility estimates were cumulated over respondents to provide an average estimate of the importance of each welfare concern [39].

#### Relative importance values

The relative importance values were calculated for each issue from the difference that each issue made to the total of all the utility values for each respondent. This difference was the range in the issue’s utility values and was calculated as a percentage of the issue’s relative range, resulting in a set of issue importance values that add to 100 percent. The relative importance values are ratios, so an issue with a relative importance of 40% is twice as important as one with a relative importance of 20%. The relative importance values for all the respondents were used to calculate mean importance values for each issue, which were then ranked in order from the highest to lowest importance [39].

#### Statistical analyses

Because there were only four people above 56 years of age, they were added to the group of people aged 46–55 years to make one group of > 46 years. Multivariate analysis of variance was used to assess the significance of the demographic factors on each issue. It was used to calculate least square means and standard errors of Importance Values. The general linear model included industry involvement, gender, education and geography as fixed factors, and age, length of involvement, and knowledge or transport and slaughter as covariates. The Anderson Darling test was used to examine the residuals for normal distribution, the Levene’s test for equality of variance and Fisher’s pairwise comparisons were used to discriminate between individual means post hoc. Where the Anderson Darling tests indicated that residuals were not normal, data were transformed to log_10_ or Box Cox, using optimal lamda, before analysis. Probability values were considered significant at <0.05.

Calculations of utility estimates and relative importance values were performed using Sawtooth Software version 8.4.8. All other analyses were performed using Minitab version 17 [Minitab 17 Statistical Software (2010). State College, PA: Minitab, Inc. (www.minitab.com)]

## Results

The questionnaire was started by 943 respondents. Of these, 332 dropped out after the question asking what industry involvement they had (presumably because they did not fit into any category), 180 dropped out after reading the instructions, 28 dropped out during the paired comparison questions, three after other issues, one after the last system question. Although 399 respondents finished the survey online, not all of them had utility values, and only 267 respondents had answered the demographic, ACA rating and importance questions, and customized paired questions contributing to the utility value.

The most common role of stakeholders was in livestock transport (Table 2), which represented 33%, and the second most common was in livestock production research or a teaching role, which both represented 27% of respondents. Only 2.2% of respondents were not involved in any way, and few were involved in an environmental protection role related to livestock, and none in a veterinary role or in livestock slaughter. Government roles relating to livestock production, livestock farmers, and livestock welfare research or advocacy were all represented (12.7%, 11.2% and 7.9% respectively).

**Table 2:** Demographic characteristics of respondents, who were stakeholders in the animal transportation and slaughter sectors, to a questionnaire on the major welfare issues in the Chinese livestock transport and slaughter industry, as defined in an initial expert meeting (n = 267).

Demographics	Numbers of respondents (%)
**Role in livestock transport and slaughter**	
Livestock production research or teaching role	72 (27.0)
Veterinary role	0 (0)
Government role relating to livestock production	34 (12.7)
Environmental protection role related to livestock	16 (6.0)
Livestock welfare research or advocacy	21 (7.9)
Livestock farming	30 (11.2)
Livestock transport	88 (33.0)
Livestock slaughter	0 (0)
Not involved	6 (2.2)
**Gender**	
Male	164 (61.9)
Female	96 (36.2)
Other	5 (1.9)
**Age (years)**	
18–25 years	105 (39.3)
26–35 years	97 (36.3)
36–45 years	47 (17.6)
46–55 years	14 (5.24)
56–65 years	2 (0.75)
65 years or more	2 (0.75)
**Geographical location**	
North China	15 (5.7)
Northeast China	4 (1.5)
East China	9 (3.4)
Central China	9 (3.4)
Southwest China	3 (1.1)
Northeast China	15 (5.7)
South China	202 (76.5)
Throughout China	5 (1.9)
Outside of China	2 (0.8)
**Education**	
University postgraduate degree	65 (24.6)
University undergraduate degree	105 (39.8)
Technical or trades college	27 (10.2)
High school	23 (8.7)
Middle school	40 (15.1)
Other	4 (1.5)
**Experience (years)**	
Less than a year	63 (24.0)
1–5 years	97 (36.9)
More than 5 years	103 (39.1)
**Knowledge of livestock transport**	
Very high level	9 (3.4)
High level	26 (9.9)
Neither a high nor low level	147 (55.7)
Low level	72 (27.3)
Very low level	10 (3.8)
**Knowledge of livestock slaughter**	
Very high level	13 (4.9)
High level	31 (11.7)
Neither a high nor low level	130 (49.2)
Low level	78 (29.6)
Very low level	12 (4.6)

### Stakeholders’ demographics

Almost two-thirds of respondents were male. Most respondents lived in South China, but there was representation from all the other regions in China. In relation to their highest educational level, nearly 40% had a university degree and a further 25% had a postgraduate degree, making this a comparatively well-educated sample of the population. Most of the respondents were experienced in their field, with fewer than 25% having less than one-year’s experience in their work. Most self-reported that their knowledge of livestock transport and slaughter was at neither a high nor a low level; alternatively, they indicated that it was at a low level.

### Importance values of the issues

The issue respondents considered most important was stunning procedure, first for cattle and sheep, then pigs and poultry (Tables 1 and S3). This was supported by a high rating of the importance of achieving and maintaining unconsciousness during slaughter. The issues respondents considered next most important were journey quality, reducing stress and maintaining animal comfort, livestock workers’ experience and attitudes, overcrowding, and type of vehicle. Considered less important than these were the loading facilities and journey length. The issues respondents considered least important were the pre-slaughter accommodation and the method and body part used for catching chickens.

### Utility values within issues

The differences in utility values within issues defines the relative acceptability of each practice for respondents in terms of animal welfare (Table 2). Major differences, over and above obvious and expected responses, are described as follows. In relation to stunning of cattle and sheep, the most acceptable procedure for welfare was electrical stunning before slaughter, and the least was hitting the animal on the head with a hard object; the latter was perceived to be worse than not stunning at all. A similar perception was evident for pigs and poultry, and carbon dioxide stunning was considered marginally worse than electrical stunning in terms of the animal welfare impact. For cattle and sheep, use of the penetrating captive bolt was perceived to be more acceptable than the percussive captive bolt. Heat stress during transport to slaughter was perceived to be less acceptable than cold stress. Open-sided vehicles were perceived to be slightly more acceptable than semi-closed vehicles, with closed-sided vehicles much less acceptable than either of these. In relation to duration of transport, journeys of less than 3 hours were perceived to be most acceptable, then 3–6 hours; journeys of more than 6 hours were least acceptable.

### Demographic effects

As respondents’ self-reported knowledge of transport increased, their rating of the acceptability of many issues increased (Table 3). Similarly, as respondents’ self-reported length of involvement in the industry increased, they increased their rating of the acceptability of several issues (Table 4).

**Table 3:** Effects of the knowledge of respondents (n = 267), who were stakeholders in the animal transportation and slaughter sectors, about transport on their ascribed utility values, which measure the perceived importance of different issues for animal welfare, when the relationship was examined by linear regression in a general linear model. The relationship is reported as a regression coefficient, SE coefficient and probability (P) value of the regression being significant.

	Coefficient	Standard Error of the coefficient	P-value
Livestock being able to stand up	0.287	0.107	0.007
Livestock remaining unconscious	0.323	0.145	0.027
Livestock transport workers with a high level of experience	0.464	0.219	0.035
Less than 3 hours journey to slaughterhouse	0.018	0.008	0.022
Comfortable temperature	0.243	0.114	0.034
Comfortable journey with no evidence of bruising or vomiting	0.280	0.117	0.018
Stunning poultry and pigs electrically	0.299	0.107	0.005
Stunning cattle and sheep electrically	0.279	0.128	0.030

**Table 4:** Significant (P< 0.05) effects of the duration of involvement of respondents, who were stakeholders in the animal transportation and slaughter sectors, with the industry on the mean utility values of issues, which measure the perceived importance of different issues for animal welfare, when the relationship is examined by linear regression in a general linear model. The relationship is reported as a regression coefficient, SE coefficient and probability (P) value of the regression being significant.

	Coefficient	Standard Error of the coefficient	P-value
Catching fully developed chickens manually by the legs and feet	0.188	0.091	0.039
Livestock remaining unconscious	0.441	0.153	0.004
Livestock being able to stand up	0.336	0.112	0.003
Stunning cattle and sheep electrically	0.365	0.134	0.007

#### Stakeholder involvement

Respondents in the livestock production teaching and research role rated stunning of cattle and sheep electrically and livestock remaining unconscious throughout the slaughter process as more acceptable, and not stunning pigs and poultry less acceptable, compared to livestock transporters (Table 5). They also, in conjunction with those in a government role and livestock farmers, rated catching poultry manually in the dark as more acceptable than livestock transporters.

**Table 5:** Significant (P< 0.05) effects of type of industry involvement of stakeholders, who were stakeholders in the animal transportation and slaughter sectors, on the mean utility values of different levels, which measure the strength of preference for animal welfare. High values indicate high acceptability on animal welfare grounds. There were no veterinary or livestock slaughterer respondents, and a small number of respondents that did not align themselves with any of the stated industries were excluded.

	Livestock production research/ teaching role	Government role	Environmental protection role related to livestock	Livestock welfare research/ advocacy	Livestock farming	Livestock transport	F value (d.f. 5, 227)	SED^†^	P-value
Poultry caught manually in the dark	0.67^a^	0.69^a^	0.65^a^^b^	0.66^a^^b^	0.69^a^	0.63^b^	2.67	0.0187	0.02
Poultry and pigs not stunned before slaughter	-0.75^b^	-0.40^a^^b^	-0.28^a^^b^	-0.45^a^^b^	-0.32^a^^b^	-0.01^a^	2.7	0.3077	0.021
Cattle and sheep electrically stunned before they are slaughtered	1.47^a^	1.04^a^^b^	0.88^a^^b^	1.40^a^^b^	1.27^a^^b^	0.84^b^	2.3	0.2667	0.032
Livestock remain unconscious throughout the slaughter process	1.42^a^	1.17^a^^b^	0.67^a^^b^	0.97^a^^b^	1.22^a^^b^	0.63^b^	2.3	0.3031	0.029

a,b: means with different superscripts differ significantly (P < 0.05) according to Fisher’s pairwise comparison.

^†^ Standard Error of the Difference between two means

### Other demographic factors

Better educated stakeholders (with a university degree) perceived stunning poultry and pigs by carbon dioxide to be more important for animal welfare than those educated only to high school or a technical college level (Table 6). Similarly, those with a postgraduate degree perceived electrical stunning of poultry and pigs to be more important for animal welfare than those educated only to a school or a technical college level. Women perceived transporting livestock by an open-sided vehicle to be more important for animal welfare than men (mean utility values 0.46 and 0.096, respectively, SED = 0.157, P = 0.04).

**Table 6:** Significant (P< 0.05) effects of level of education of stakeholders, who were stakeholders in the animal transportation and slaughter sectors, on the mean utility values of issues, which measure the perceived importance of different issues for animal welfare. High values indicate high acceptability.

	University postgraduate degree	University undergraduate degree	Technical or trades college	High school	Middle school	Other	F value (d.f. 5, 227)	SED^†^	P-value
Poultry/pigs electrically stunned before slaughter	1.19^a^	0.91^a^^b^	0.51^b^	0.46^b^	0.55^b^	1.68^a^	2.56	0.2060	0.018
Poultry/pigs stunned with CO_2_ before slaughter	0.66^a^^b^	0.77^a^	0.18^b^	0.07^b^	0.24^a^^b^	-1.46^c^	3.7	0.2376	0.004

a,b: means with different superscripts differ significantly (P < 0.05) according to Fisher’s pairwise comparison

^†^ Standard Error of the Difference between two means

## Discussion

In this study of Chinese stakeholders in the livestock transport and slaughter industries, stunning for cattle and sheep was rated as the most important animal welfare concern, amongst the different options given, followed by stunning for pigs and poultry. In contrast, the animal welfare concern considered least important was the method of catching chickens. This suggests that Chinese stakeholders were more concerned about slaughter issues than transport issues. This is in accordance with a recent study of the attitudes of Asian students of veterinary medicine and animal science towards slaughter and transport of livestock, in which the students in China were more concerned about slaughter issues than transport issues [40]. A greater concern for the slaughter process, rather than transport, is also in accordance with views in Australia towards slaughter standards in Indonesia, where, in particular, the absence of stunning has caused major concern amongst the public [41]. However, a survey in Australia showed that road and ship transport were of greater concern than pre-slaughter stunning to stakeholders in the Australian livestock industries [20]; this is probably because the pre-slaughter stunning is the norm in Australian abattoirs. Concern about slaughter practices in China has been heightened recently by the link between slaughter of poultry in markets and the emergence of bird flu (H7N9) [42]. This occurred initially in 2013 and by January 2017 had caused the deaths of 79 people, with 192 people infected [42]. In some provinces markets have been required to end the slaughter of poultry [43].

It was expected that government officials would demonstrate greater concern for welfare issues associated with international trade as some livestock and poultry products have been refused by the European Union and the USA due to welfare concerns [11]. However, this was not observed in our results, perhaps because the officials were not dealing directly with international trade. Most respondents were either involved in livestock and poultry production or in livestock teaching and researching roles, and most (75%) had more than one year’s experience in their work. As respondents’ level of education increased they rated stunning before slaughter to be more important, and self-reported professional knowledge and experience were also positively associated to the importance attributed to stunning. In China, the relationship between meat quality and welfare prior to slaughter has been widely reported in academic literature [44, 45, 46, 47]. Despite this, the maintenance of good conditions during transport and slaughter is often neglected [48]. In the absence of any transport legislation or animal welfare legislation generally, and a strong market imperative to grow the livestock industries, there is little incentive to improve welfare at this point in the animal’s life [28].

Chinese consumers are showing more concern about livestock and poultry products’ quality and safety than previously [11]. Issues relating to unsafe food have occurred frequently in the country, and this has had an impact on public health. It has also negatively impacted on the national economy as products are imported at high cost (e.g. milk powder) from overseas as they are considered to be safer [49]. As food safety is currently of significant concern to consumers in China, one way to encourage good conditions for livestock during transport and slaughter is to emphasize the links between improved conditions and food safety [11]. In addition, according to a recent survey of Chinese public preferences for pork, nearly 77% of consumers prefer to purchase meat produced under good welfare conditions even if it costs more [11].

The survey also highlighted some of the aspects of transport and slaughter that are related to the geographical conditions in the country. For example, respondents in this study rated open-sided vehicles more acceptable in terms of animal welfare than semi-closed and closed-sided vehicles. Livestock and poultry are generally transported at a high density in China, and in the summer transport may occur in high temperatures, which will cause heat stress and sometimes even death [48]. Good ventilation during transport is considered one of the most important factors, which may improve the meat quality of the carcass [48]. Most (77%) of the respondents were from South China where the ambient temperatures are often high. Ventilation is generally better in open-sided than closed-sided vehicles [48], and good ventilation will reduce the temperature to which animals are exposed during transport [50]. Conversely in the northern Europe where there are often cooler conditions and lower stocking densities, closed-sided vehicles are usually preferred due to the ability to control ventilation, and, consequently avoid the chilling of livestock, e.g. [51] for chickens. Journey length was not considered very important but this may have been because the options were <3, 3–6 and > 6 hours, which reflects the fact that in China pigs and chickens may be raised relatively close to their point of slaughter. There are however many exceptions where livestock are transported long distances across this large country, mainly from the inland regions to provide meat for consumers in the east coast cities [28].

### Stakeholder involvement

Significant differences were observed between the respondents in different roles in livestock transport and slaughter, in particular between transport workers and livestock production teachers/researchers. The latter found stunning for livestock and catching the poultry in the dark to be more acceptable than did the transport workers. It has been observed previously that those involved in an aspect of a production process are more likely to rate it as more important [20, 29]. However, the livestock transport workers in this survey did not rate transport issues as more important than slaughter issues. This may be because transport workers in China are not directly involved with the animals, but are organizing the transport. They are also generally not well educated, and so unlikely to be well informed about animal welfare [10]. The emphasis on stunning by those respondents that were well educated, including the teachers/researchers, may reflect their understanding of its benefit in alleviating suffering at the end of life, which transporters may not appreciate. This suggests that it is important to give teachers/researchers the opportunity to evaluate livestock transport and slaughter systems in China.

### Gender effects

The similarity of female and male responses to this survey (in which responses differed significantly for only one issue: transporting livestock in open-sided vehicles thought to be more acceptable by women) is consistent with a recent study in which males and females in China had no difference in attitudes on animal welfare issues [52]. However, reviewing mainly Western literature, Herzog [53] found females more sympathetic to animal welfare and less accepting of animals’ suffering. In a cross-cultural study, it was found that only in countries with a high Gender Empowerment Measure could women to express their concern for animals [54], which is reported to be greater than that in men [53,55–59]. In China, the degree of gender empowerment is high at 0.164, with a world ranking of 37^th^ in the world [60], which suggests that women would express greater concern. However, the Confucian belief that all animals should be treated well may equally influence men and women [61], and the communist influence in the 20^th^ C also encouraged equality between men and women [62]. Together these two facts may explain why there was little difference seen in responses between men and women in this study.

## Limitations of the study

The benefits of virtual snowballing include the ability to collect data from a large number of people rapidly and inexpensively, and false reporting may be reduced by the anonymity of this sampling technique [63, 64]. In contrast, there is significant potential for selection bias with this sampling technique and it is not possible to estimate response rates. It has, however, been reported that internet surveys using this method give similar results to traditional sampling methods and can make valuable contributions to research [64, 65]. We acknowledge that the respondents probably represented a well-educated and geographically limited sector of the stakeholders in this industry. It was also clear that many people started the survey but did not complete it, which may have been due to their eligibility as stakeholders, the time taken to complete it, or their lack of interest in the subject matter. This is not uncommon in adaptive conjoint analysis surveys, which require respondents to think carefully, but it suggests a bias, that those that completed the survey were more likely to be those more interested in, and able to provide, detailed evaluation of livestock welfare. There was evidence of this since well-educated respondents issued a higher utility value to stunning procedures, which may be due to their being more aware of the welfare benefits these provide. An over-representation of well-educated respondents is valuable for the survey since reliable answers are likely to be obtained, but it does not necessarily represent the majority view. Less well-educated respondents may be more likely to rely on intuition, which may favour more emotive issues such as avoiding causing pain to livestock.

The survey required stakeholders to have access to the internet, which only 52% of the Chinese population has [66]. Since it is mainly the older generation and the rural poor that lack access, it was expected that most stakeholders in this industry would have access, but it must be acknowledged that some of those most regularly in contact with these rural industries did not have access to, or regularly use, the internet.

Due to the unbalanced geographical distribution of respondents, with 76% of the respondents from the South of China, the distribution is unlikely to represent the overall perspectives of all Chinese people. People from different regions in China may have different attitudes because of different levels of economic and social development [9]. In addition, the absence of livestock slaughterers and veterinarians in the sample may have influenced the results, as people in these roles have very specific and important functions and may have different perceptions of the importance of welfare issues in China. Similarly, less well-educated farmers and those that are actively engaged in, rather than just organising, livestock transport, were not adequately represented, and they may have had a different, more pragmatic view on welfare problems during livestock transport and slaughter. However, the importance of welfare enhancement is arguably restricted to the high value supply chains for livestock products. There is generally much less opportunity for less well-educated farmers to obtain increased prices for value-added products, such as welfare-enhanced livestock products, compared with the larger scale suppliers to the major food chains, particularly if they are exporting their product to the EU [67]. Nevertheless, it is important to obtain views from a widespread set of representatives in the livestock sector. Future studies could use community-focused group meetings to elicit responses directly from stakeholders that are hard to reach. These would need to provide incentives for participation, including veterinarians, as the benefit to them is not as obvious as, for example, teachers/researchers, who need to have a detailed understanding of how the industry functions. Farmers are particularly hard to reach, especially as they are older than city dwellers on average, with 55% over 40, or they are rural migrant workers, working both in the cities and on their farms and expanding at about 4% per year [68]. In the latter case, much of the daily agricultural management of the small plots allocated to rural dwellers is done by older members of families. These would be very hard to reach in participatory research. At the same time social science research could gather public opinion on the welfare concerns which have been identified, and whether they would welcome animal products that come from high welfare systems addressing these issues, even if they cost more [69]. The combination of providing information to the public on welfare issues and allowing them to express their opinions is gathering momentum in solving animal welfare problems [69].

It is also possible that our initial focus group meeting failed to identify some of the biggest problems, and therefore stakeholders were unable to select the ones that they believed to be most important. Although water injecting was raised as a potential issue, but omitted because it has been declared illegal in recent years, many Chinese researchers have recently reported it as a current problem [48, 70, 71, 72, 73]. The extent of the practice is unknown but it has obvious welfare concerns. It was not investigated in this study due to it being an issue of great sensitivity in China, its illegality and concerns about the influence this would have on potential responses and the risk that such an investigation might pose for respondents. Future studies should investigate the prevalence of this practice and stakeholder and public attitudes towards it. It is also possible that some of our descriptions of the levels of each issue were potentially leading, by describing attitudes as good or poor, for example, for which benign or malevolent would have been more appropriate.

## Conclusions

This study reports the perceived major welfare concerns for livestock during transport and slaughter in China, as identified by a sample of stakeholders in the industry that rated issues that had been proposed by a focus group. Although the stakeholders may not be entirely representative of the industry, there was reasonable agreement on the most serious welfare issues. Slaughter-related issues represented the major concerns, in common with previous surveys addressing transport and slaughter in Asia. This may reflect an increased importance placed on the critical moments of taking life, as compared to other potentially more chronic concerns. Livestock transporters rated catching poultry manually in the dark as less acceptable and not stunning pigs and poultry as more acceptable than many other stakeholder groups, demonstrating some differences between stakeholder groups in the perception of welfare issues. Future research should address key welfare issues identified here with a broader range of stakeholders and examine the potential impacts of improvement of welfare on farmers in particular.

## Supporting information

S1 Table Australian version of the questionnaire. (DOCX)

S2 Table Chinese version of the questionnaire. (DOCX)

S3 Table Mean and standard deviations of the utility and importance values. (DOCX)
