# Supplementary material for: Perception of animal welfare issues during Chinese transport and slaughter of livestock by a sample of stakeholders in the industry
Source: PLoS One. 2018 Jun 22;13(6):e0197028. doi: 10.1371/journal.pone.0197028 (PMC6014659; doi:10.1371/journal.pone.0197028)
Supplement: S1 Table — (DOCX) [file pone.0197028.s001.docx]

**Animal Welfare Issues in China Questionnaire**

**The purpose of this survey is to provide a more in-depth understanding of perceived animal welfare issues in livestock transport and slaughter in China. The information obtained will be used to assist the Chinese livestock industry in progressing animal welfare. Your participation is entirely voluntary and your answers anonymous. Your assistance and your contribution to furthering animal welfare and the interests of the Chinese livestock industry will be greatly appreciated.**

- **I confirm that I am over 18 years old, have read and understood the information above about this project, consent to participating in this research and would like to continue with the survey.**

**The first section of this questionnaire consists of 10 brief questions which are demographic questions. These help us to describe the population we are studying. Your answers are anonymous and will only be reported in general terms (for example, 45% of the study population was female).**

**Your responses to this questionnaire are anonymous but if you do not feel comfortable answering a question please select "Prefer not to answer".**

1. How are you currently involved in livestock transport and slaughter in China?

(please choose only one option)

- Livestock production research or teaching role
- Veterinary role
- Government role relating to livestock production
- Environmental protection role related to livestock
- Livestock welfare research or advocacy
- Livestock farming
- Livestock transport
- Livestock slaughter
- Not involved

1. You identify as......

- Male
- Female
- Other

1. What age group are you in?

- 18-25 years
- 26-35 years
- 36-45 years
- 46-55 years
- 56-65 years
- 66 or more years

1. What is the highest level of education that you have attained?

- Primary school
- Secondary school
- University undergraduate degree
- University postgraduate degree
- No formal schooling
- Technical or trades college
- Other

1. How long have you been involved with the livestock industries in China?

- Less than a year
- 1-5 years
- More than 5 years

1. In what geographical location in China do you work?

- South China
- Throughout China
- Outside of China
- Southwest China
- Southeast China
- Northeast China
- Northwest China
- West China
- East China
- North China

1. How would you rate your knowledge of livestock transport in China?

- Very high level of knowledge
- High level of knowledge
- Neither a high nor low level of knowledge
- Low level of knowledge
- Very low level of knowledge

1. How would you rate your knowledge of livestock slaughter in China?

- Very high level of knowledge
- High level of knowledge
- Neither a high nor low level of knowledge
- Low level of knowledge
- Very low level of knowledge

**In this section you will be asked about different livestock management scenarios and, in your opinion, how acceptable they are in relation to animal welfare.**

**Please consider only the immediate impact of each scenario on the animals’ welfare, regardless of the associated practical and economic factors.**

**"Animal welfare" means how an animal is coping with the conditions in which it lives. An animal is in a good state of welfare if it is healthy, comfortable, well nourished, safe, able to express innate behaviour, and if it is not suffering from unpleasant states such as pain, fear, and distress.**

Please rate the different levels of the following animal welfare issues in relation to the acceptability of the animal welfare outcome resulting from that each scenario.

1. Experience of livestock transport workers

|  | Acceptability of the animal welfare outcome resulting from each scenario | | | | |
| --- | --- | --- | --- | --- | --- |
|  | Very unacceptable | Somewhat unacceptable | Neither unacceptable or acceptable | Somewhat acceptable | Very acceptable |
| Livestock transport workers with little or no experience |  |  |  |  |  |
| Livestock transport workers with a moderate level of experience |  |  |  |  |  |
| Livestock transport workers with a high level of experience |  |  |  |  |  |

1. Attitude of livestock transport workers

|  | Acceptability of the animal welfare outcome resulting from each scenario | | | | |
| --- | --- | --- | --- | --- | --- |
|  | Very unacceptable | Somewhat unacceptable | Neither unacceptable or acceptable | Somewhat acceptable | Very acceptable |
| Livestock transport workers with a good attitude |  |  |  |  |  |
| Livestock transport workers with a reasonable attitude |  |  |  |  |  |
| Livestock transport workers with a poor attitude |  |  |  |  |  |

1. Method of catching chickens

|  | Acceptability of the animal welfare outcome resulting from each scenario | | | | |
| --- | --- | --- | --- | --- | --- |
|  | Very unacceptable | Somewhat unacceptable | Neither unacceptable or acceptable | Somewhat acceptable | Very acceptable |
| Poultry are caught manually in the dark |  |  |  |  |  |
| Poultry are caught manually in the light |  |  |  |  |  |

1. Part of body used to catch fully developed chickens

|  | Acceptability of the animal welfare outcome resulting from each scenario | | | | |
| --- | --- | --- | --- | --- | --- |
|  | Very unacceptable | Somewhat unacceptable | Neither unacceptable or acceptable | Somewhat acceptable | Very acceptable |
| Fully developed chickens are manually caught by the head or wings or tail |  |  |  |  |  |
| Fully developed chickens are manually caught by the legs and feet |  |  |  |  |  |

1. Loading facilities

|  | Acceptability of the animal welfare outcome resulting from each scenario | | | | |
| --- | --- | --- | --- | --- | --- |
|  | Very unacceptable | Somewhat unacceptable | Neither unacceptable or acceptable | Somewhat acceptable | Very acceptable |
| Animals are loaded using facilities with a steep  loading ramp slope, a slippery floor or sharp turns |  |  |  |  |  |
| Animals are loaded using facilities with a gently sloping loading ramp, a non-slip floor and no sharp turns |  |  |  |  |  |

1. Type of road vehicle

|  | Acceptability of the animal welfare outcome resulting from each scenario | | | | |
| --- | --- | --- | --- | --- | --- |
|  | Very unacceptable | Somewhat unacceptable | Neither unacceptable or acceptable | Somewhat acceptable | Very acceptable |
| Livestock are transported by closed-sided vehicle |  |  |  |  |  |
| Livestock are transported by open-sided vehicle |  |  |  |  |  |
| Livestock are transported by semi-closed vehicle |  |  |  |  |  |

1. Overcrowding

|  | Acceptability of the animal welfare outcome resulting from each scenario | | | | |
| --- | --- | --- | --- | --- | --- |
|  | Very unacceptable | Somewhat unacceptable | Neither unacceptable or acceptable | Somewhat acceptable | Very acceptable |
| Livestock being transported are not able to stand up due to overcrowding |  |  |  |  |  |
| Livestock being transported are able to stand up |  |  |  |  |  |

1. Journey length

|  | Acceptability of the animal welfare outcome resulting from each scenario | | | | |
| --- | --- | --- | --- | --- | --- |
|  | Very unacceptable | Somewhat unacceptable | Neither unacceptable or acceptable | Somewhat acceptable | Very acceptable |
| Journey to slaughterhouse is less than 3 hours |  |  |  |  |  |
| Journey to slaughterhouse is 3-6 hours |  |  |  |  |  |
| Journey to slaughterhouse is more than 6 hours |  |  |  |  |  |

1. Maintenance of animal comfort during transportation (temperature)

|  | Acceptability of the animal welfare outcome resulting from each scenario | | | | |
| --- | --- | --- | --- | --- | --- |
|  | Very unacceptable | Somewhat unacceptable | Neither unacceptable or acceptable | Somewhat acceptable | Very acceptable |
| The temperature during transport causes heat stress to the livestock |  |  |  |  |  |
| The temperature during transport causes cold stress to the livestock |  |  |  |  |  |
| The temperature during transport is comfortable for the livestock |  |  |  |  |  |

1. Maintenance of animal comfort during transportation (provision of rest and water)

|  | Acceptability of the animal welfare outcome resulting from each scenario | | | | |
| --- | --- | --- | --- | --- | --- |
|  | Very unacceptable | Somewhat unacceptable | Neither unacceptable or acceptable | Somewhat acceptable | Very acceptable |
| During long distance transport livestock are provided with a stop for water |  |  |  |  |  |
| During long distance transport livestock are NOT provided with a stop for water |  |  |  |  |  |

1. Stress during journey

|  | Acceptability of the animal welfare outcome resulting from each scenario | | | | |
| --- | --- | --- | --- | --- | --- |
|  | Very unacceptable | Somewhat unacceptable | Neither unacceptable or acceptable | Somewhat acceptable | Very acceptable |
| During transport livestock have a comfortable journey with no evidence of bruising or vomiting |  |  |  |  |  |
| During transport livestock have an uncomfortable and stressful journey and there is some evidence of bruising or vomiting |  |  |  |  |  |
| During transport livestock have a stressful journey and there is significant bruising and mortality of livestock |  |  |  |  |  |

**Slaughter Issues**

1. Pre-slaughter accommodation

|  | Acceptability of the animal welfare outcome resulting from each scenario | | | | |
| --- | --- | --- | --- | --- | --- |
|  | Very unacceptable | Somewhat unacceptable | Neither unacceptable or acceptable | Somewhat acceptable | Very acceptable |
| Cattle, pigs and sheep are provided with 6 hours or more rest and water before slaughter |  |  |  |  |  |
| Cattle, pigs and sheep are provided with no rest or water before slaughter |  |  |  |  |  |

1. Stunning procedures for Poultry and Pigs

|  | Acceptability of the animal welfare outcome resulting from each scenario | | | | |
| --- | --- | --- | --- | --- | --- |
|  | Very unacceptable | Somewhat unacceptable | Neither unacceptable or acceptable | Somewhat acceptable | Very acceptable |
| Poultry and pigs are electronically stunned before they are slaughtered |  |  |  |  |  |
| Poultry and pigs are stunned with carbon dioxide before they are slaughtered |  |  |  |  |  |
| Poultry and pigs are stunned by hitting them on the head with a hard object |  |  |  |  |  |
| Poultry and pigs are NOT stunned before they are slaughtered |  |  |  |  |  |

1. Stunning procedures for cattle and sheep

|  | Acceptability of the animal welfare outcome resulting from each scenario | | | | |
| --- | --- | --- | --- | --- | --- |
|  | Very unacceptable | Somewhat unacceptable | Neither unacceptable or acceptable | Somewhat acceptable | Very acceptable |
| Cattle and sheep are electronically stunned before they are slaughtered |  |  |  |  |  |
| Cattle and sheep are stunned by hitting them on the head with a hard object |  |  |  |  |  |
| Cattle and sheep are stunned with a percussive captive bolt |  |  |  |  |  |
| Cattle and sheep are stunned with a penetrating captive bolt |  |  |  |  |  |
| Cattle and sheep are NOT stunned before they are slaughtered |  |  |  |  |  |

1. Achievement of unconsciousness

|  | Acceptability of the animal welfare outcome resulting from each scenario | | | | |
| --- | --- | --- | --- | --- | --- |
|  | Very unacceptable | Somewhat unacceptable | Neither unacceptable or acceptable | Somewhat acceptable | Very acceptable |
| Livestock remain unconscious throughout the slaughter process |  |  |  |  |  |
| Livestock regain consciousness after stunning during the slaughter process |  |  |  |  |  |

**In this next section, you will be presented with two livestock management scenarios. Please consider whether the scenarios are different in terms of their immediate impact on animal welfare.**

**If you think that animal welfare does not differ between the two scenarios, please choose “not important”. If you think there is a difference between the two scenarios, then use the scale to show how important you think the difference is.**

**(Please assume that all other aspects of the livestock management are acceptable)**

*Example (10 different scenarios using the questions above will be presented)*

|  | The importance of the difference between the two practices is in terms of the livestock welfare | | | | |
| --- | --- | --- | --- | --- | --- |
|  | Not important | Neither important or unimportant | Somewhat important | Moderately important | Extremely important |
| Fully developed chickens are manually caught by the head or wings or tail  --- instead of--- Fully developed chickens are manually caught by the legs and feet |  |  |  |  |  |

**In this next section, you will be presented with two combinations of livestock management scenarios, with one combination presented on the LEFT side of the screen and the other on the**

**RIGHT side.**

**You will be asked which combination you think is better in terms of animal welfare.**

**(Please assume that all other aspects of the livestock management are acceptable and consider just the immediate impact of each procedure on the animal welfare)**

*Example (10 different scenarios using the questions above will be presented)*

| Poultry are caught in the light  Livestock transport workers with a poor attitude | OR | Poultry are caught in the dark  Livestock transport workers with a good attitude |
| --- | --- | --- |

| Strongly prefer left scenario | Somewhat prefer left scenario | Prefer neither left nor right scenario | Somewhat prefer right scenario | Strongly prefer right scenario |
| --- | --- | --- | --- | --- |
|  |  |  |  |  |

**Please type a number between 0 and 100 where 0 means "This scenario is definitely NOT acceptable from an animal welfare point of view" and 100 means ""This scenario is very acceptable from an animal welfare point of view"**

How acceptable is this scenario from an animal welfare point of view?

*Example Scenario (6 different scenarios using the questions above will be presented)*

Livestock transport workers with a poor attitude

Fully developed chickens are manually caught by the legs and feet

Poultry are caught manually in the light

**Are there any other welfare issues associated with livestock transport and slaughter in China that you believe are important but were not covered in this questionnaire? If so can you please give details below? Thank you**

**______________________________________________________________________________________________________________________________________________________________________________________________________________________________________________________**
