# Supplementary material for: Perception of animal welfare issues during Chinese transport and slaughter of livestock by a sample of stakeholders in the industry
Source: PLoS One. 2018 Jun 22;13(6):e0197028. doi: 10.1371/journal.pone.0197028 (PMC6014659; doi:10.1371/journal.pone.0197028)
Supplement: S2 Table — (DOCX) [file pone.0197028.s002.docx]

本人确认，本人已年满18岁，已阅读并理解上述关于该项目的信息，同意参与本次调研，并愿意继续此调查问卷。

男性

女性

其他

18-25 岁

26-35 岁

36-45 岁

46-55 岁

56-65 岁

66 岁或以上

市区（市中心区）

郊区 (位于离市中心10公里远或更远的郊区的住宅区)

区域城市 (人口 > 50,000)

乡下小镇 (人口 = 5 万或更少)

农村 (居住在面积大于 20 英亩的一片土地上)

半农村 (居住在面积大于 1 英亩但小于 20 英亩的一片土地上)

其他

不愿意回答

我的职业是家畜生产员/出运前管理员/屠宰者

我的职业是家畜运输员

我的职业是家畜屠宰者

我的职业是政府部门的家畜生产员和管理员

我的职业是环境保护者

我的职业是一家兽医机构中与家畜有关的工作人员

我的职业是畜牧生产员/管理研究人员和/或学术人员

我的职业是家畜福利研究人员、学术人员和/或拥护者

知识水平非常高

知识水平高

知识水平既不高也不低

知识水平低

知识水平非常低

小学

中学

大学本科

研究生

无正规教育

专科院校或贸易学院

其他

少于1年

1-5 年

5 年以上

家畜生产研究或教学

不相关

兽医

政府部门与家畜生产有关的工作人员

与家畜有关的环境保护工作人员

家畜福利研究员或拥护者

家畜饲养员

家畜运输员

家畜屠宰者

整个中国

国外

华北地区

中国东南地区

华南地区

华东地区

中国西北地区

中国东北地区

中国西南地区

华西地区

家畜运输工人，有经验

家畜运输工人，经验较少或没有经验

家畜运输工人，经验水平适度

家畜运输工人，经验水平高

运输工人的态度

家畜运输工人，态度良好

家畜运输工人，态度合理

家畜运输工人，态度差

家禽捕捉环境

夜间人工捕捉家禽

日间人工捕捉家禽

捉住鸡的身体部分

完全发育的鸡由人工捉住鸡头、鸡翅膀或鸡尾巴捕到

完全发育的鸡由人工捉住鸡腿和鸡爪捕到

装货设备

家畜采用带有装卸陡坡、光滑地板或急转弯的设备装载

家畜采用带有装卸缓坡、防滑地板和没有急转弯的设备装载

道路车辆类型

家畜采用侧面封闭的车辆运输

家畜采用侧面敞开的车辆运输

家畜采用半封闭的车辆运输

过度拥挤

运输的家畜能够站立

运输的家畜由于过度拥挤无法站立

行程长度

距离屠宰场的行程不到3小时

距离屠宰场的行程为3-6小时

距离屠宰场的行程超过6小时

在运输期间保持动物舒适度（温度）

运输期间的温度使家畜受到热压

运输期间的温度使家畜受到冷压

运输期间的温度使家畜感到舒适

在运输期间保持动物舒适度（提供休息时间和水）

在长途运输过程中，停下来让家畜饮水

在长途运输过程中，没有停下来让家畜饮水

行程中的压力

运输过程中，家畜所在环境舒适，没有瘀伤或呕吐现象

运输过程中，家畜所在环境不舒适并且存在压力，部分家畜出现瘀伤或呕吐现象

运输过程中，家畜所在环境存在压力，家畜出现严重的瘀伤或呕吐现象

宰前适应

宰前6小时或更长时间内让牛、猪和羊得到休息和饮水

宰前没有让牛、猪和羊得到休息和饮水

家禽和猪的击昏程序

宰前电昏家禽和猪

宰前使用二氧化碳使家禽和猪昏倒

使用硬物击打家禽和猪的头部使其昏倒

宰前没有使家禽和猪昏倒

牛和羊的击昏程序

宰前电昏牛和羊

使用硬物击打牛和羊的头部使其昏倒

使用敲击式手枪型机械式致昏器使牛和羊昏倒

使用穿透性手枪型机械式致昏器使牛和羊昏倒

宰前没有使牛和羊昏倒

实现昏迷

在整个屠宰过程中，家畜始终处于昏迷状态

在整个屠宰过程中，家畜被击昏后仍然有意识

请输入一个位于[ ]和[ ]之间的数字，[ ]是指“从动物福利视角来看，这种情况是绝对不被认可的”，[ ]是指“从动物福利视角来看，这种情况是完全认可的”

从动物福利视角来看，这种情况被认可的程度如何？

[ ] *代替* [ ]

在家畜福利方面这两种情况间差异的重要性

不重要

有点重要

重要

非常重要

极其重要

这两个组合中，哪个代表更好的动物福利情况？

或者

特别倾向于左边

比较倾向于左边

中立

比较倾向于右边

特别倾向于右边

各种情况造成的动物福利结果的可接受性

根本无法接受

无法接受

有点无法接受

既不是无法接受，也不是可以接受

稍微可以接受

可以接受

完全可以接受

中国家畜运输和屠宰的动物福利问题

家畜屠宰和运输的动物福利问题

此页发生一个错误。

请修复此错误，并重试。

必须回答。

[ ]

最小检查数为[ ]。

[ ]

最大检查数为[ ]。

[ ]

回答内容必须在[ ]和[ ]之间。

[ ]

回答内容必须是一个数字。

[ ]

回答内容必须包含至少[ ]个字符。

[ ]

回答内容必须包含超过[ ]个字符。

[ ]

无其他采访内容，不得再使用此登录名。

您的调查问卷已提交。

谢谢。

无效登录。

访问被拒绝。

每个回答内容都必须是唯一的。

[ ]

请排列[ ]选项。

[ ]

不得同时选择“以上都不是”和其他选项。

[ ]

回答内容必须总和为[ ]。

其当前总和为[ ]。

[ ]

相同项不得选择两次。

[ ]

您选择的部分特点组合在一起不可行。

禁止组合已突出显示。

请重新考虑。

[ ]

您所选择的一项或多项价格发生变化。

请复核价格。

此调查问卷正在更新。

请几秒后重试。

产品不得同时选为最佳和最差

此调查问卷需要使用启用了 JavaScript 功能的浏览器。

请打开浏览器的JavaScript功能，然后再次输入此调查问卷。

此调查问卷是为了更深入了解已经发现的中国家畜运输和屠宰的动物福利问题。

所获信息将用于帮助中国家畜行业解决正在处理的动物福利问题。

您的参与完全自愿，您的答案是不记名的。

非常感谢您对动物福利事业和中国家畜行业利益的支持和贡献。

您目前如何参与中国的家畜运输和屠宰工作？

（请选择一个选项）

此调查问卷的第一部分包括一些简单的个人信息问题。

这些有助于我们描述我们正在研究的人群。

您的答案是不记名的，并且将按照笼统的形式进行报道（例如，研究人群的45%是女性）。

如果您认为某个问题不适合回答，请跳过此问题。

请确定……

您属于哪个年龄组？

您获得的最高教育程度是什么？

您参与中国家畜行业的工作有多长时间？

您的工作位于中国哪个地理位置？

您如何评价自己在中国家畜运输方面具备的知识？

您如何评价自己在中国家畜屠宰方面具备的知识？

在这一部分，您需要回答有关不同家畜管理情况的问题，以及您认为这些情况从动物福利视角来看被接受的程度如何。

只考虑每种情况对动物福利的直接影响，不管任何相关的实际因素和经济因素。

“动物福利”是指动物如何应付其所处的环境。

动物健康、舒适、营养良好、安全、能够表达天生行为，并且如果没有遭受痛苦状态，如疼痛、恐惧和难受，就说明动物状态良好。

从动物福利视角来看，您认为以下各种家畜管理情况被接受的程度如何？

在下一部分中，您将会看到两种家畜管理情况。

请考虑这两种情况是否在对动物福利的直接影响上存在不同之处。

如果您认为这两种情况的动物福利没有差异，请选择“不重要”。

如果您认为这两种情况存在差异，请通过数值范围体现差异的重要性。

（假设家畜管理的所有其他方面都是可以接受的）

在下一部分中，您将会看到家畜管理情况的两种组合，屏幕左右两侧各呈现一种组合。

您需要回答哪种组合的动物福利更好。

（假设家畜管理的所有其他方面都是可以接受的，并且只考虑每种情况对动物福利的直接影响）

您认为还有其他与中国家畜运输和屠宰有关的重要的而此调查问卷中未包含的福利问题吗？

如果还有，请在下方详述？

谢谢

感谢您参与本次调研。

非常感谢您的帮助，您的回答内容已记录在案。
