# Supplementary material for: Perception of animal welfare issues during Chinese transport and slaughter of livestock by a sample of stakeholders in the industry
Source: PLoS One. 2018 Jun 22;13(6):e0197028. doi: 10.1371/journal.pone.0197028 (PMC6014659; doi:10.1371/journal.pone.0197028)
Supplement: S3 Table — (DOCX) [file pone.0197028.s003.docx]

**S3 Appendix. Mean and standard deviations of the utility and importance values**

|  |  |  |
| --- | --- | --- |
| Average Utilities (Zero-Centered Diffs) | Average Utilities | Standard Deviation |
| Little experience | -44.98520 | 34.92062 |
| Moderate experience | 5.56463 | 23.54783 |
| High experience | 39.42056 | 34.29611 |
| Good attitude | 29.96533 | 30.66580 |
| Reasonable attitude | 12.87870 | 26.58592 |
| Poor attitude | -42.84403 | 34.02642 |
| In dark | -18.22276 | 44.40629 |
| In light | 18.22276 | 44.40629 |
| Head, wings or tail | -29.98318 | 32.05665 |
| Legs and feet | 29.98318 | 32.05665 |
| Poor loading facilities | -17.19216 | 45.81191 |
| Good loading facilities | 17.19216 | 45.81191 |
| Closed-sided | -26.92487 | 40.03241 |
| Open-sided | 16.46607 | 41.04448 |
| Semi-closed | 10.45880 | 32.67889 |
| Can stand up | 34.29886 | 37.45124 |
| Cannot stand up | -34.29886 | 37.45124 |
| <3hrs | 27.73928 | 38.27278 |
| 3-6hrs | 0.55500 | 17.45622 |
| >6hrs | -28.29428 | 35.45075 |
| Heat stress | -34.76629 | 34.07495 |
| Cold stress | -20.49188 | 28.81541 |
| Comfortable temp | 55.25817 | 37.72822 |
| Stop and water provided | 26.60483 | 43.02722 |
| Stop and water NOT provided | -26.60483 | 43.02722 |
| Comfortable journey | 53.09659 | 38.60940 |
| Uncomfortable and stressful journey with bruising and V+ | -12.81723 | 23.01936 |
| Stressful journey with significant bruising and mortality | -40.27936 | 38.12140 |
| 6hrs rest and water | 28.28164 | 36.36249 |
| No rest or water | -28.28164 | 36.36249 |
| Electronic stunning | 27.07206 | 38.64269 |
| Carbon dioxide stunning | 24.80387 | 45.67749 |
| Blunt trauma hit | -38.68677 | 34.61177 |
| No stunning | -13.18916 | 56.91120 |
| Electronic stunning | 36.34297 | 44.63089 |
| Blunt trauma hit | -28.38304 | 31.72711 |
| Percussive captive bolt | -4.51323 | 38.01048 |
| Penetrating captive bolt | 9.84696 | 35.67229 |
| No stunning | -13.29365 | 63.80722 |
| Remain unconscious | 28.92020 | 51.45938 |
| Regain consciousness | -28.92020 | 51.45938 |
|  |  |  |
| Average Importances | Average Importances | Standard Deviation |
| Transport worker experience | 7.05996 | 2.40744 |
| Attitudes of transport workers | 6.46872 | 2.37824 |
| Poultry catching environment | 5.51407 | 3.23462 |
| Catching chickens | 5.37982 | 2.29343 |
| Loading facilities | 5.84950 | 2.87050 |
| Vehicle type | 6.18729 | 2.66573 |
| overcrowded | 6.26108 | 2.56497 |
| Length of journey | 5.69899 | 2.72144 |
| Temperature | 7.72339 | 2.33606 |
| Rest and water | 6.18647 | 2.66968 |
| Stress during journey | 7.78381 | 2.84174 |
| Pre-slaughter accommodation | 5.58737 | 2.53837 |
| Stunning pigs and poultry | 8.51828 | 2.71315 |
| Stunning for cattle and sheep | 8.63518 | 2.96611 |
| Achieving unconsciousness | 7.14609 | 3.27766 |
